# CDE++: Learning Categorical Data Embedding by Enhancing Heterogeneous Feature Value Coupling Relationships

**DOI:** 10.3390/e22040391

**Published:** 2020-03-29

**Authors:** Bin Dong, Songlei Jian, Ke Zuo

**Affiliations:** College of Computer, National University of Defense Technology, Changsha 410000, China; dongbin09@nudt.edu.cn (B.D.); zuoke@nudt.edu.cn (K.Z.)

**Keywords:** categorical data, data embedding, heterogeneous couplings, hybrid clustering strategy, autoencoder, clustering, classification

## Abstract

Categorical data are ubiquitous in machine learning tasks, and the representation of categorical data plays an important role in the learning performance. The heterogeneous coupling relationships between features and feature values reflect the characteristics of the real-world categorical data which need to be captured in the representations. The paper proposes an enhanced categorical data embedding method, i.e., CDE++, which captures the heterogeneous feature value coupling relationships into the representations. Based on information theory and the hierarchical couplings defined in our previous work CDE (Categorical Data Embedding by learning hierarchical value coupling), CDE++ adopts mutual information and margin entropy to capture feature couplings and designs a hybrid clustering strategy to capture multiple types of feature value clusters. Moreover, Autoencoder is used to learn non-linear couplings between features and value clusters. The categorical data embeddings generated by CDE++ are low-dimensional numerical vectors which are directly applied to clustering and classification and achieve the best performance comparing with other categorical representation learning methods. Parameter sensitivity and scalability tests are also conducted to demonstrate the superiority of CDE++.

## 1. Introduction

Categorical data with finite unordered feature values are ubiquitous in machine learning tasks, such as clustering [1,2] and classification [3,4]. Most machine learning algorithms are built for numerical data based on algebraic operations, such as k-means and SVM, which cannot be directly used for categorical data. These algebraic machine learning algorithms will be applicable for categorical data only if we embed the categorical data into numerical vector space. However, learning numerical representations of categorical data is not a trivial task since the intrinsic characteristics in categorical data need to be captured in embeddings.

As stated in [5], the hierarchical couplings relationship (i.e., correlation and dependency) between feature values in categorical data is a crucial characteristic which should be mined sufficiently. The sophistic couplings between feature values also reflect the correlations between features. Take the simple dataset in Table 1 as an example. It is intuitive that the value (short for feature value) Female of feature Gender is highly coupled with the value Liberalarts of feature Major. Similarly, The value Engineering in feature Major is strongly coupled with the value Programmer in feature Occupation. Thus, the relation between feature Gender and Major could be expressed by a semantic cluster, i.e., {Female, Liberalarts}, as well as feature Major and Occupation by {Engineering, Programmer}. These value clusters which may contain multiple values reflect the heterogeneous couplings in categorical data. Moreover, the feature value clusters are also coupled with each other in both same and different granularities. These high-level couplings are heterogeneous and therein exists both linear and nonlinear relationships.

For most learning tasks, the more relevant information (i.e., the hierarchical couplings) the categorical data embeddings captures, the better performance it has. However, besides CDE [5], other representation learning methods could capture only limited or none of the couplings in categorical data. Generally, existing methods fall into two categories: the embedding-based method and the similarity-based method. Typical embedding methods, e.g., 1-hot encoding and Inverse Document Frequency (IDF) encoding [6,7], transform categorical data to numerical data by some encoding schemes directly. But these methods treat features independently and ignore the couplings between feature values. Also, several similarity-based methods, e.g., ALGO (clustering ALGOrithm), DILCA (DIstance Learning for Categorical Attributes), DM (Distance Metric), COS (COupled attribute Similarity) [8,9,10,11], take value couplings into consideration. However, these methods do not take feature value intrinsic clusters and couplings between clusters into account so that their representation capacities are limited for categorical data.

Learning the heterogeneous hierarchical couplings in categorical data is not a trivial task. There are short of work representing hierarchical couplings in categorical data so far. To our knowledge, our previous work CDE (Categorical Data Embedding) [5] is the first work focusing on hierarchical couplings mining and categorical data representing. Compared with other existing representation methods, it gets relatively better performance. However, CDE can only capture homogeneous value clusters through single clustering strategy and linear correlation between value clusters through principal component analysis which limits its performance in complex categorical data.

To address the above issues, we propose an enhanced Categorical Data Embedding method, i.e., CDE++, which can capture heterogeneous feature value relationships in categorical data. In value couplings learning phase, we use mutual information and margin entropy to learn the interactions of features and feature values. To learn the value clusters couplings, we design a hybrid clustering strategy to get heterogeneous value clusters from multiple aspects. Then the Autoencoder is adopted on these value cluster indicator matrices to obtain lower-dimensional value embeddings which can capture complex nonlinear relationships between value clusters. We finally concatenate the value embeddings to generate an expressive object representation. In this way, CDE++ can capture the intrinsic data characteristic of categorical data in the expressive numerical embeddings which largely facilitate the following learning tasks.

The contributions of this work are summarized as follows:

- By analyzing the hierarchical couplings in categorical data, we propose an enhanced Categorical Data Embedding method (CDE++), which could capture heterogeneous feature value coupling relationships in each level.
- We adopt mutual information and margin entropy to capture the couplings between features and design a hybrid clustering strategy to capture more sophisticated and heterogeneous value clusters in the low level. CDE++ implements different metric-based clustering methods, including density-based clustering method and hierarchical clustering method, with various clustering granularities from different perspectives and semantics.
- We utilize Autoencoder to learn the complex and heterogeneous value cluster couplings in the high level. With this, CDE++ maps the original value representation into a low-dimensional space, while learning both linear and nonlinear value cluster coupling relationships.
- We empirically prove the superiority of CDE++ through both supervised and unsupervised learning tasks. Experiment results show that (i) CDE++ significantly outperforms the state-of-the-art methods and their variants in both clustering and classification. (ii) CDE++ is insensitive to its parameters and thus has stable performance. (iii) CDE++ is scalable w.r.t. the number of data instances.

The rest of this paper is organized as follow. Related work is discussed in Section 2. We introduce the proposed method, i.e., CDE++, in Section 3. Experiments setup and results analysis are provided in Section 4. We conclude this work in Section 5.

## 2. Related Work

Existing representation learning algorithms broadly fall into two categories: (i) embedding-based representation which represents each categorical object by a numerical vector, (ii) similarity-based representation which uses object similarity matrix to represent the categorical object.

### 2.1. Embedding-Based Representation

Embedding-based representation, which is the most widely used in categorical data representation, generates a numerical vector to represent each categorical object. A popular embedding method called 1-hot encoding translates each feature value to a zero-one indicator vector [6]. It first counts the values of one feature $f_{i}$ as $|V_{i}|$. Then the value in the feature is represented by a 1-hot $|V_{i}|$-dimension vector, where ‘1’ corresponds to the value entry and ‘0’ to the others. 1-hot encoding treats each value equally and ignores the instinct couplings of real datasets. Our previous work CDE [5] is a state-of-the-art embedding-based representation which makes use of coupling relationships of data sets. However, the method could not exploit heterogeneous coupling relationships comprehensively due to its clustering method and the limits of nonlinear relationship mining. This method uses a dimension reduction method, such as the principal component analysis (PCA) [12], to alleviate the curse of the dimensionality issue. IDF encoding is another popular embedding-based representation method [7], and it utilizes the probability-weighted amount of information (PWI), which is calculated based on the value frequency, to represent each value. IDF-encoding learns couplings between values from the occurrence perspective, accordingly, its ability of mining intrinsic coupling relationships of data set is very limited. The method in [13] has the same goal as our work, which is to learn transforms categorical data to numerical representations for categorical data. The main difference between the method in [13] and our method is that they need class labels while our method is an unsupervised method.

Embedding-based representation methods are also used for textual data, and there are several effective embedding methods such as Skim-gram [14], latent semantic indexing (LSI) [15], latent Dirichlet allocation (LDA) [16], as well as some variants of them in [17,18,19]. Granular Computing paradigm [20,21,22] is an embedding method which is powerful especially when dealing with non-conventional data such as graphs, sequences, text documents. However, the embedding representation for textual data is significantly different from categorical data since categorical data is structured, whereas textual data is unstructured. Thus, we do not detail these embedding methods here.

### 2.2. Similarity-Based Representation

Similarity-based representation methods utilize an object similarity matrix to represents categorical data. The inspiration of several similarity-based methods comes from learning couplings of categorical data. For instance, ALGO [8] first takes advantage of conditional probability in a pair of values to describe the value couplings; DILCA [9] learns a context-based distance between feature values to capture feature couplings; DM [10] incorporates the frequency probabilities and feature weighting to mining couplings of the feature. COS [11] grasps couplings from two aspects, i.e., inter-feature and intra-feature. The above similarity measures learn feature couplings by pair-values. However, they could not obtain comprehensive couplings since the value clusters and the couplings therein are not considered. Moreover, the similarity methods are inefficient because they require to calculate and store the object similarity matrix.

There are several embedding methods that utilize similarity matrix to optimize their embedding representations [23,24]. However, the performance of these embedding methods depends heavily on the underlying similarity methods.

## 3. Method of CDE++

### 3.1. Learning Process of CDE++

**Figure 1:**
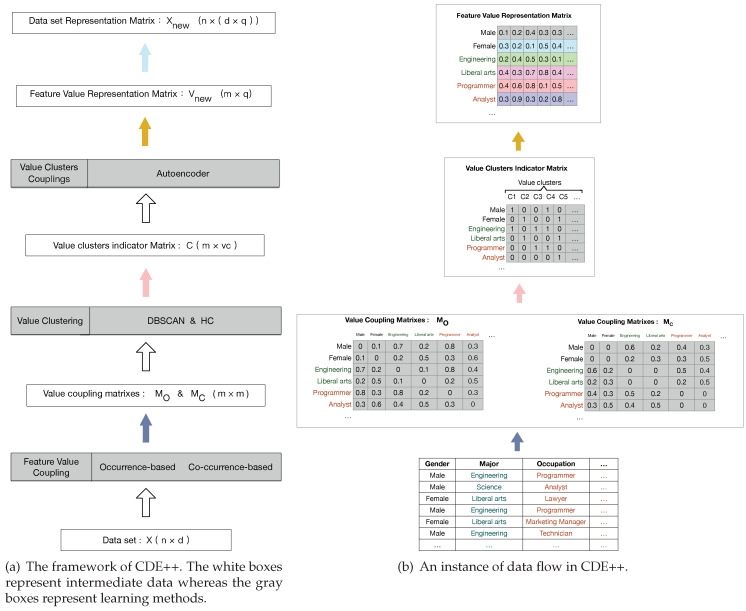
Overview of CDE++.

We aim to rebuild the categorical data set so as to make it more convenient for the following learning tasks. Figure 1a illustrates the framework of our enhanced Categorical Data Embedding Learning method (CDE++). The gray boxes in Figure 1a represent a series of learning methods, whereas the white boxes consist of a certain amount of intermediate data for our representation rebuilding. Figure 1b is an instance of data flow in CDE++. The notations are illustrated in Table 2.

As shown in Figure 1, we first construct the value couplings matrices by occurrence-based and co-occurrence-based value coupling method, which can capture the interactions between values. Then, we learn value clusters by hybrid clustering strategy with multiple granularities. After obtaining the value clusters, we learn the couplings between value clusters by the deep neural network, Autoencoder, for the value representation. Finally, we obtain the object representation by concatenating the value vectors for the following learning tasks.

### 3.2. Preliminaries

Consider a dataset *X* with *n* objects, that is, $X=\{x_{1},x_{2},...,x_{n}\}$, where each object $x_{i}$ is described by *d* categorical features, and the features belong to $F=\{f_{1},f_{2},...,f_{d}\}$. Each feature $f_{i}$ has a finite set of values $V_{i}=\left\{v_{i1},v_{i2},...\right\}$. Moreover, the values from different features has no intersection such that the number of total feature values is $\left|V\right|=\sum_{i=1}^{d}\left|V_{i}\right|$, denoted as *m*.

For better describing how to calculate the joint probability of two values $v_{i}$ and $v_{j}$, we need to introduce some symbols. Let $f^{i}$ denotes the feature that $v_{i}$ belongs to, and let $v_{x}^{f}$ denotes the value in feature *f* of object *x*. Let $p(v_{i})$ denotes the probability of $v_{i}$ that calculated by its occurrence frequency. Thus, the joint probability of $v_{i}$ and $v_{j}$ is

(1) $$p\left(v_{i},v_{j}\right)=\frac{|v_{x}^{f^{i}}=v_{i}\cap v_{x}^{f^{j}}=v_{j}|}{n},\forall x\in X.$$

The normalized mutual information, denoted as NMI, is a measurement of the mutual dependence between two vectors [25]. When we observe one vector, the information of the other vector that we can obtain can be quantified by NMI. Accordingly, the relation between two features $f_{a}$ and $f_{b}$ could defined as

(2) $$\rho \left(f_{a},f_{b}\right)=\frac{2I(f_{a},f_{b})}{H\left(f_{a}\right)+H\left(f_{b}\right)},$$

where $I(f_{a},f_{b})$ is the relative entropy of joint distribution and marginal distribution, and it is written in

(3) $$I\left(f_{a},f_{b}\right)=\sum_{v_{i}\in V_{f_{a}}}\sum_{v_{j}\in V_{f_{b}}}p\left(v_{i},v_{j}\right)log\frac{p(v_{i},v_{j})}{p\left(v_{i}\right)p\left(v_{j}\right)}.$$

$H(f_{a})$ and $H(f_{b})$ are the marginal entropies of feature $f_{a}$ and $f_{b}$, respectively. The marginal entropy of the specific feature can be described by

(4) $$H\left(f\right)=-\sum_{v_{i}\in V_{f}}p\left(v_{i}\right)log\left(p\left(v_{i}\right)\right),f\in f_{a},f_{b}.$$

### 3.3. Learning Value Couplings

The value couplings are learned to reflect the intrinsic relationship between feature values. As we used in the previous work [5], which is proved effective and intuitional. The relation between values has two aspects: on the one hand, the occurrence frequency of one value is influenced by others; on the other hand, one value could be influenced by its pair value because of their co-occurrence relationship in one objects. For capturing the value couplings based on occurrence and co-occurrence, two coupling functions and their corresponding relation matrices (m×m) are constructed, respectively.

The occurrence-based value coupling function is $\xi_{o}\left(v_{i},v_{j}\right)=\rho \left(f^{i},f^{j}\right)\times \frac{p(j)}{p(i)}$, which represents the occurrence frequency of $v_{i}$ influenced by $v_{j}$. In this function, the NMI of two features works as a weight. After constructing the coupling function, the occurrence-based relationship matrix $M_{o}$ is constructed by:

(5) $$M_{o}=\left\{\begin{array}{ccc} \xi_{o}\left(v_{1},v_{1}\right) & \cdots & \xi_{o}\left(v_{1},v_{m}\right)\\ \vdots & \ddots & \vdots \\ \xi_{o}\left(v_{m},v_{1}\right) & \cdots & \xi_{o}\left(v_{m},v_{m}\right) \end{array}\right\}$$

The co-occurrence-based value coupling function is $\xi_{c}\left(v_{i},v_{j}\right)=\frac{p(v_{i},v_{j})}{p(v_{i})}$, which indicates the co-occurrence frequency of value $v_{i}$ influenced by value $v_{j}$. Note that $f^{i}$ and $f^{j}$ will never be equal since it is impossible for two values owned by the same feature to co-occur in one object. Thus, the co-occurrence-based relationship matrix $M_{c}$ is designed as follow:

(6) $$M_{c}=\left\{\begin{array}{ccc} \xi_{c}\left(v_{1},v_{1}\right) & \cdots & \xi_{c}\left(v_{1},v_{m}\right)\\ \vdots & \ddots & \vdots \\ \xi_{c}\left(v_{m},v_{1}\right) & \cdots & \xi_{c}\left(v_{m},v_{m}\right) \end{array}\right\}$$

The two matrices could be treated as new representations of value couplings based on occurrence and co-occurrence, respectively. Moreover, they could be applied in the following values clustering.

### 3.4. Hybrid Value Clustering

To capture the value clusters from different perspectives and semantics, we cluster the feature values in different granularities and use the new representation ($M_{o},M_{c}$) as the input of the clustering algorithm. To make the cluster results more robust and reflect the data characteristics more precisely, we choose a hybrid clustering strategy, which combines the clustering results of DBSCAN (Density-Based Spatial Clustering of Applications with Noise) and HC (Hierarchical Clustering).

The motivation we use the hybrid clustering strategy is as follows: (i) The metric of DBSCAN is density-based, whereas HC is a partition-based method like K-means. So when we combine the cluster results of the two clustering methods, we can obtain the comprehensive value clusters, which is crucial for capturing the intrinsic data characteristics. (ii) DBSCAN has excellent performance for both convex data sets and non-convex data sets, whereas K-means is not suitable for non-convex data sets. HC can also solve the non-spherical datasets that K-means can not solve. (iii) DBSCAN is not sensitive to noisy points, which means DBSCAN is stable. Consequently, our hybrid clustering strategy suitable for majority data sets; meanwhile, it has a better clustering result.

DBSCAN contains a pairwise parameter τ(eps,MinPts), where eps represents the maximum radius of circles centered on cluster cores, and MinPts represents the minimum number of objects in the circle. HC only has one parameter *K*, which means the number of clusters likes K-means. Therefore, for clustering with different granularities, we set parameters $\left\{\tau_{1},\tau_{2},...,\tau_{o}\right\}$ and $\left\{\tau_{1}^{{}^{\prime }},\tau_{2}^{{}^{\prime }},...,\tau_{c}^{{}^{\prime }}\right\}$ for $M_{o}$ and $M_{c}$ clustering with DBSCAN respectively. Likewise, we set parameters $\left\{k_{1},k_{2},...,k_{o^{{}^{\prime }}}\right\}$ and $\left\{k_{1}^{{}^{\prime }},k_{2}^{{}^{\prime }},...,k_{c^{{}^{\prime }}}^{{}^{\prime }}\right\}$ for clustering with HC.

Parameter selection. In HC clustering, the strategy of choosing *K* is demonstrated in Algorithm 1. Instead of giving a fixed value, we use another proportion factor ε to decide the maximum cluster number as shown in Steps (3-12) of Algorithm 1. We remove those tiny clusters with only one value from the indicator matrix. When the number of removed clusters is larger than $\frac{k}{\varepsilon }$, we stop increasing *K*, whose initial value is 2. In DBSCAN clustering, for a specific τ(eps,MinPts), the parameter eps and MinPts are selected based on k-distance graph. For a given *k*, the k-distance function is mapping each point to its k-th nearest neighbor. We sort the points of the clustering database in descending order of their k-distance values. Furthermore, we set eps to the first point in the first “valley” of the sorted k-distance graph, and we set MinPts as value *k*. The value *k* is same to the parameter *K* of HC. The parameter selection is following [26].

After clustering, we get four clustering indicator matrices to represent the clustering results. The clustering indicator matrix of $\left(M_{o},DBSCAN\right)$ is denoted as $C_{o_{d}}$, the size of which is m×o. Likewise, other indicator matrices are $\left\{C_{o_{h}},C_{c_{d}},C_{c_{h}}\right\}$ with size $\left\{m\times c,m\times o^{{}^{\prime }},m\times c^{{}^{\prime }}\right\}$. Finally, we concatenate the four indicator matrices into one indicator matrix, denoted as *C*, which contains the comprehensive information of value clusters. Similar to $M_{o}$ and $M_{c}$, *C* could also be regarded as a new representation of feature values based on value couplings and value clusters.

### 3.5. Embedding Values by Autoencoder

Deep Neural Network (DNN) is the hottest topic in machine learning because of its ability in feature extraction. Each middle layer in DNN has the ability of feature learning; it is a self-learning process without any prior knowledge.

After constructing the value clusters indicator matrix *C*, which contains comprehensive information, we further learn the couplings between the value clusters. Meanwhile, it requires to build a concise but meaningful value representation. It is intuitional for us to use DNN for value clusters couplings learning, and we use Autoencoder to handle this in unsupervised circumstance. The simple function of Encoder and Decoder are as follows:

Encoder:code=f(x),

$$Decoder:x^{{}^{\prime }}=g\left(code\right)=g\left(f\left(x\right)\right).$$

The Encoder is used to learn low-dimension representation code of the input *X*. Each layer of the Encoder learns the feature and features couplings of Input *X*, therefore, code contains the complete information of *X*. The Decoder is implemented to reconstruct X from its input, i.e., code. The training process of Autoencoder is minimizing the loss function Loss[x,g(f(x))]. After training, the code will contain the feature couplings of *X* and convey similar information with *X* as well.

The Autoencoder makes it possible for us to capture the heterogeneous value clusters couplings and obtain a relatively low dimension values representation. In our method, we train the Autoencoder by using the value clusters indicator matrix *C* as the input. Furthermore, we use the Encoder to calculate a new values representation matrix $V_{new}$ in m×q. The column size *q* is determined by $|o+c+o^{{}^{\prime }}+c^{{}^{\prime }}|$ (denoted by vc) and hidden factor λ which will be discussed in Section 4.5. The new value representation $V_{new}$ would convey the information of value clusters *C* as well as the clusters couplings, which is considered as a concise but meaningful value representation.

### 3.6. The Embedding for Objects

The final step is to model the objects embedding after we get values representation from Autoencoder. The general function is presented as

(7) $$x_{new}=\Omega \left(v_{1}^{x},v_{2}^{x},\cdots ,v_{d}^{x}\right),v\in V_{new}.$$

The function Ω in Equation (7) could be customized to suit for learning task in the following. We concatenate the new values from $V_{new}$ to generate the new objects embedding.

The main procedures of CDE++ are presented in Algorithm 1. Algorithm 1 has three inputs, that is, the data set *X*, the factor of drop redundancy value clusters ε, the hidden factor of Autoencoder λ. The algorithm mainly consists of four steps. The first step is to calculate $M_{o}$ and $M_{c}$ based on occurrence and co-occurrence value coupling function. Then CDE++ utilizes hybrid clustering strategy to cluster values with $M_{o}$ and $M_{c}$. The parameter ε is used to control the clustering results and determines the time to terminate the clustering process. In the third step, the algorithm uses Autoencoder to learn the couplings of value clusters and generates the concise but meaningful value embedding. The parameter λ is a hidden factor of the input dimension and output dimension of Encoder, which indicates the ratio of dimension compression. Finally, CDE++ embeds objects in the data set by concatenating the value embedding.
**Algorithm 1** Object Embedding (X,ε,λ)**Input:** Dataset *X*, Parameters ε and λ**Output:** the new representation of *X* ($X_{new}$)1:Generate $M_{o}$ and $M_{c}$2:Initialize C=ϕ3:**for**$M\in \{M_{o},M_{c}\}$**do**4:    **for** classifier∈{DBSCAN,HC} **do**5:        Initialize τ(eps,Minpts),k6:        Initialize r=07:        **while** $r\geq \frac{k}{\varepsilon }$ **do**8:           C=[C;classifier(M,τork)]
9:           Remove the clusters containing only one value and count as *r*10:        **end while**
11:    **end for**
12:**end for**13:Train Autoencoder14:$V_{new}=Encoder\left(C,\lambda \right)$15:$X_{new}=concatenate\;values\;from\;V_{new}$16:Return $X_{new}$

Complexity Analysis. (1) Generating value couplings matrix incurs the complexity of $O(nd^{2})$; (2) Values clustering needs the complexity of $O(m\ln m+m^{2})$; (3) The complexity of Autoencoder is O(m∗vc∗epoch), where vc and epoch are total value clusters and the iteration times respectively. (4) Generating numerical object embedding by value embeddings has the complexity of O(nd). Accordingly, the total complexity of CDE++ is $O(nd^{2}+m\ln m+m^{2}+m*vc*epoch+nd)$. In real data sets, the number of values in one feature is generally small, thus, $m^{2}$ is a little larger than $d^{2}$. Meanwhile, $m^{2}$ is not comparable with $nd^{2}$. The total number of value clusters vc is much smaller than *m* and epoch is iteration times which is manual setting. Therefore, the approximate time complexity of CDE++ is simplified as $O(nd^{2}+m*vc*\mathrm{epoch}$).

## 4. Experiments and Evaluation

### 4.1. Experiment Settings

#### 4.1.1. Data Sets

For evaluating the performance of CDE++, as Table 3 shows, fifteen real-world datasets from UCI (https://archive.ics.uci.edu/ml/datasets.php) machine learning repository are used. These datasets cover multiple areas, e.g., life, physical, game, social, computer, education, etc. Each data set has a class label as a metric, and has several features described by categorical value.

In the unsupervised K-means task, we use the whole dataset as training sets and test sets. In the supervised SVM task, we use 75% of the datasets for training sets and the rest 25% for test sets.

The detailed attributes of data sets are presented in Table 3, where {n,d,q,|C|} denotes the number of objects, features, feature values, ground-truth classes in the data set respectively.

#### 4.1.2. Baseline

In this test, CDE++ is compared with IDF encoding (denoted by “IDF”), DILCA, our previous work (i.e., the coupled data embedding denoted as “CDE”), and the widely used 1-hot coding (denoted by “1-HOT”). Moreover, to make a fair comparison, we introduce the variations of CDE and 1-HOT by replacing their last step of generating value embedding with AutoEncoder. The variations are denoted by CDE-AE and 1-HOT-AE, respectively. In CDE and its variation, the parameters are set according to its original paper. The parameters of CDE++ are mentioned in Section 4.5. We use Autoencoders with same parameters settings, as shown in Table 4.

#### 4.1.3. Evaluation Methods

The performance of learning tasks significantly depends on the data representation. The more expressive the representation is, the better the performance. To give a convincing evaluation, we feed the obtained representation into both unsupervised and supervised learning tasks. Without loss of generality, we choose K-means as the representative unsupervised learning task, whereas SVM as the representative of supervised learning tasks.

In K-means clustering, we set the number of clusters K=|C| in each data set. We use the widely used *F-score* to measure the performance. The higher the *F-score*, the better the K-means clustering performance, so as to the object representation performance. Although the datasets we used are relative balance, we choose the micro version of *F-score*. The calculation of micro *F-score* is shown below.

(8) $$precision=\frac{\sum TP_{i}}{\sum \left(TP_{i}+FP_{i}\right)},recall=\frac{\sum TP_{i}}{\sum \left(TP_{i}+FN_{i}\right)},$$

(9) $$\text{F-score}=2\frac{recall\times precision}{recall+precision},$$

where $TP_{i}$, $FP_{i}$, $FN_{i}$ are the numbers of true positive, false positive, false negative for class *i*.

For the SVM classifying, we use *Accuracy* as the performance measurement. Likewise, the higher the *Accuracy*, the better the performance of object representation.

Since the starting points of value clustering are random, we run the proposed CDE++ 10 times and feed the obtained representations into the learning tasks. Each task is repeated 10 times to get a stable result. The reported *F-score* or *Accuracy* is the average value over these 100 validations. Therefore, the robustness of evaluation results is guaranteed.

#### 4.1.4. Experimental Environment

All the experiments are conducted on the same workstation.

**Hardware environment** Macbook Pro 2016; CPU: Intel Core i7; RAM: 16 GB. Apple Inc. USA.

**Software environment** Matlab 2018b. MathWorks Inc. USA.

### 4.2. Results of Clustering

Table 3 presents the K-means clustering *F-score* of the tested methods. In thirteen out of fifteen datasets, CDE++ has the best performance, which is much better than other embedding methods. On average, CDE++ obtains approximate 16.58 %, 14.56 %, 9.10 %, 10.80 %, 13.56 %, 12.50 % improvement compared with IDF, DILCA, CDE, CDE-AE, 1-HOT, 1-HOT-AE, respectively. CDE outperforms other state-of-art representation methods due to the learning of hierarchical couplings, while CDE++ enhance the heterogeneous value relationship capturing and achieve the best performance.

### 4.3. Results of Classification

Table 5 demonstrates the *Accuracy* of SVM using the representations output by CDE, CDE-AE, 1-HOT, 1-HOT-AE, and CDE++. CDE++ performs significantly better than the first four methods, and is comparably better than 1-HOT and 1-HOT-AE. On average, CDE++ obtains approximate 12.76%, 13.55%, 10.38%, 17.3%, 5.8%, 5.11% improvement compared with IDF, DILCA, CDE, CDE-AE, 1-HOT, 1-HOT-AE, respectively. In the supervised learning task, our enhanced CDE++ could also keep a high performance than others. Therefore, based on the results above, CDE++ has generality for both unsupervised tasks and supervised tasks.

### 4.4. Ablation Study

To examine whether all the components of CDE++ is necessary, we implement the ablation study, and Table 6 shows the comparative group setting. We implement K-means clustering and SVM classification learning task using the output of objects embedding. In the implementation, (i) and (ii) use DBSCAN and HC for value clusters learning respectively, whereas (iii) uses both of them. Neither (i), (ii) nor (iii) learn value clusters couplings. (iv) uses all parts of CDE++.

Table 7 and Table 8 illustrate the K-means clustering and SVM classifying performance, respectively. Under the whole parts of CDE++, these two learning tasks obtain the highest *F-score* and *Accuracy*. Based on the ablation study, it is believed that no components can be dropped from CDE++ and the whole structure could return better objects embedding.

### 4.5. Sensitivity Test w.r.t. Parameters ε and λ

We examine the sensitivity of the performance of CDE++ w.t.r. ε and λ in this part. The first parameter ε is used to control the dimension of feature value representation before conducting Autoencoder, whereas the second parameter λ controls the dimension of feature value representation after Autoencoder. For the robustness of the test, we have selected four datasets by different level of clustering performance (i.e., F-score) for the sensitivity test. Both parameters are in the range of {2,4,6,8,10,20,40}.

**Figure 2:**
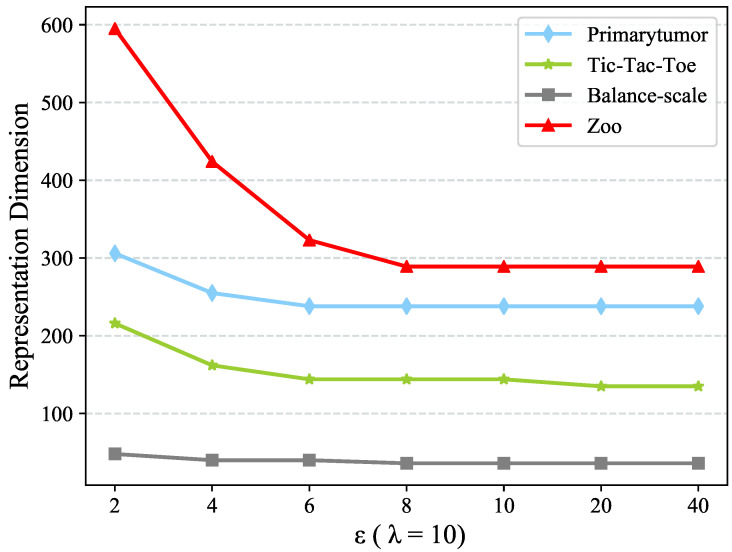
Sensitivity test w.r.t. parameter ε in term of Representation Dimension.

**Figure 3:**
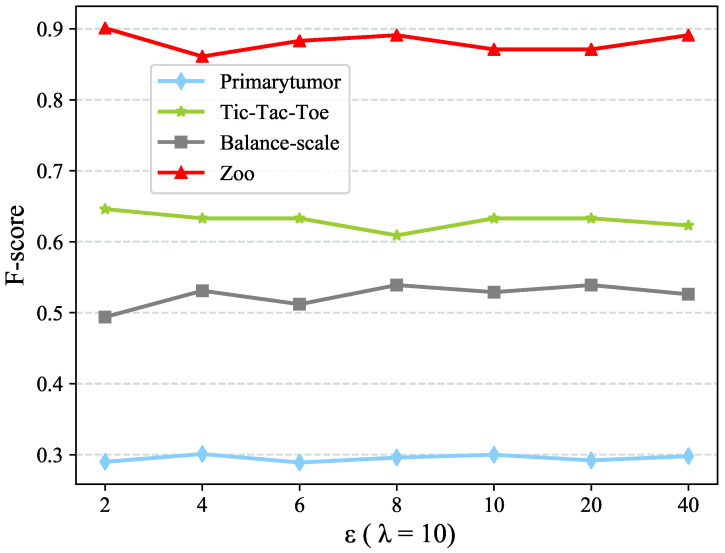
Sensitivity test w.r.t. parameter ε in term of F-score.

To test the sensitivity w.r.t. ε, we first fix λ=10. Figure 2 and Figure 3 present the dimension of objects representation and clustering performance using different ε values. The dimension of objects representation is stable when ε≥8 whereas the clustering performance is always stable. The reason why CDE++ is stable w.r.t ε is that ε only chooses some granularities of value coupling clustering. Under such granularities, the clusters with only one value have been dropped. Thus, it makes the clustering performance stable, so as to the CDE++.

**Figure 4:**
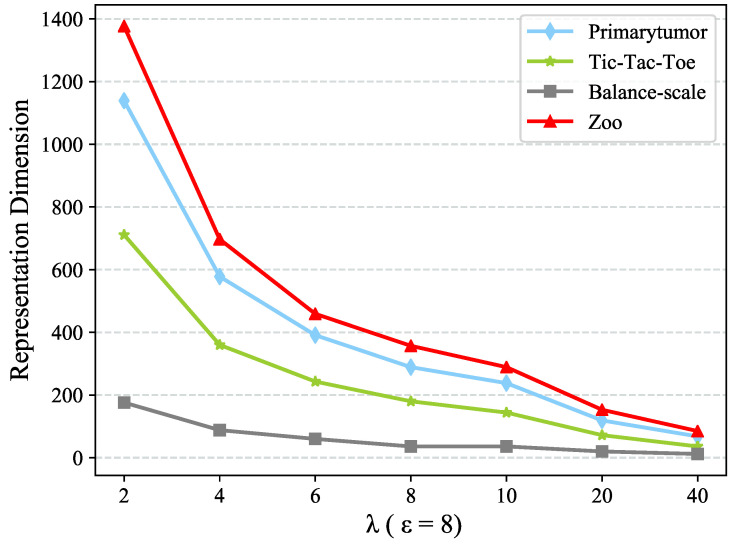
Sensitivity test w.r.t. hidden factor λ in term of Representation Dimension.

**Figure 5:**
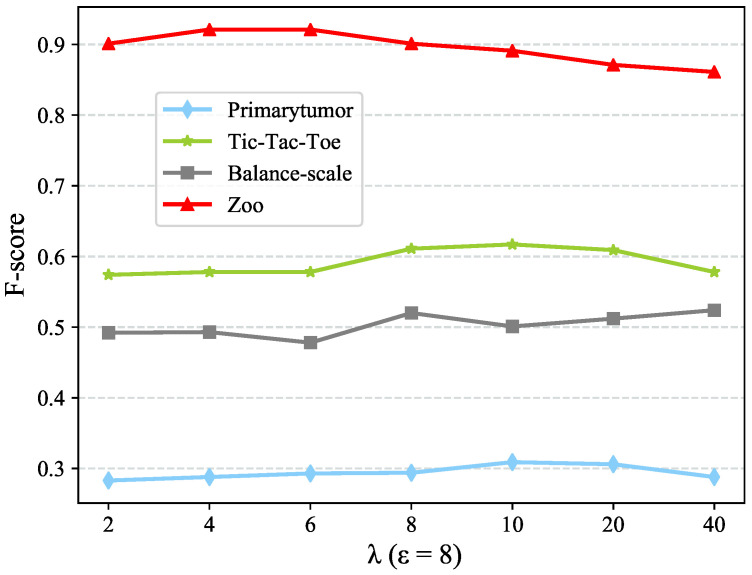
Sensitivity test w.r.t. hidden factor λ in term of F-score.

Figure 4 and Figure 5 present the dimension of objects representation and clustering performance using different λ values. Likewise, we fix ε=8 to test the sensitivity w.r.t. λ. Figure 5 shows that the clustering performance is relatively stable as a whole in the range of λ. However, the dimension of objects representation decreases as λ increases, which is illustrated in Figure 4. λ is the parameter that adjusts the ratio of the output dimension and input dimension in Autoencoder, and λ is inversely proportional to the output value representation after Autoencoder. In the value range mentioned above, though the dimension of value representation decreases, it could convey similar information in virtue of the Autoencoder algorithm. So the clustering performance would not fluctuate acutely.

Upon the sensitivity test results, we can claim that the CDE++ performance is not sensitive w.r.t. ε and λ. Moreover, we suggest ε=8 and λ=10 as a general parameters value pair.

### 4.6. Scalability Test

We split the largest dataset, i.e., Chess, in our work into five subsets, where the data size increase doubly, for the scaleup test w.r.t. data size. The subsets of Chess have six fixed features. Likewise, we synthetic data sets by varying the dimensions in [20,320] for the scalability test w.r.t. data dimension with fixed data size (e.g., 10,0 0). The feature value of the synthetic data sets is randomly chosen from {0,1}.

**Figure 6:**
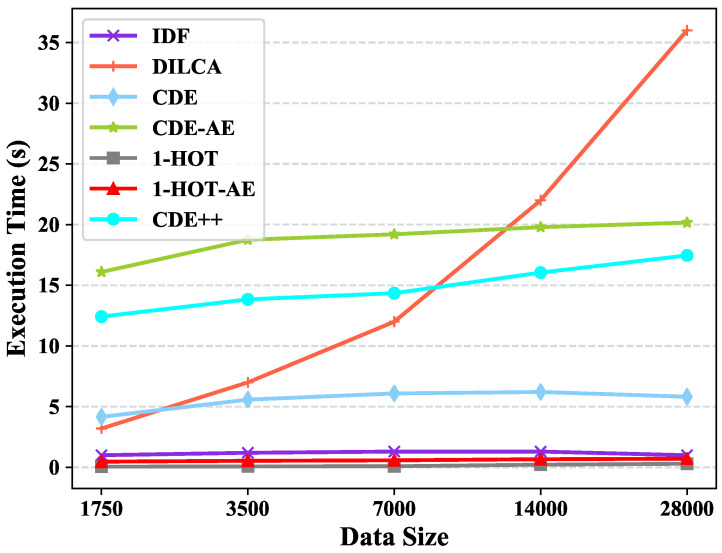
Scalability test w.r.t Data Size in term of Execution Time.

Figure 6 presents the scalability test results of the five embedding methods. As Figure 6 illustrates, the execution times increase subtly as the data set size increases. It demonstrates that the execution time of CDE++ is linear to the data size and the scalability of CDE++ w.r.t. data set size is well, while DILCA has $O(n^{2}d^{2}\log d)$.

1-HOT is the most efficient embedding method since it does not consider the couplings between feature values and just translate feature value to a 1-hot vector. The time complexity of CDE++ and CDE before learning clusters coupling are similar, since the neural network Autoencoder is more time consuming than PCA, the execution time of CDE++ is longer than CDE. When we replace the PCA of CDE with Autoencoder, the execution times increase and become even longer than CDE++.

**Figure 7:**
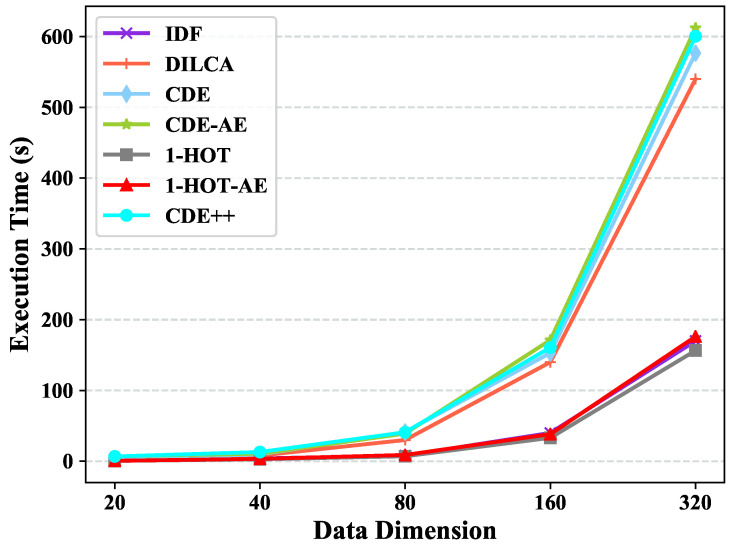
Scalability test w.r.t Data Dimension in term of Execution Time.

Figure 7 shows the execution time of the tested methods with different object dimensions. When the object dimension enlarges, the execution times of all the five methods rise up acutely. 1-HOT and 1-HOT-AE are much faster since they are simpler than other methods as introduced above. CDE++, CDE, and CDE-AE have higher and similar execution time because their complexities are quadratic functions of the feature number. Specifically, the execution time of CDE++ performed on a dataset with 10,000 objects and more than 300 features is about 10 minutes. Thus, we can say that the execution time is still acceptable in high dimension dataset embedding.

## 5. Conclusions

This paper proposes an enhanced Categorical Data Embedding method (CDE++), which aims to generate an expressive representation for complex categorical data by capturing heterogeneous feature value coupling relationships. We design a hybrid clustering strategy to capture more sophisticated and heterogeneous value clusters in the low level. We utilize Autoencoder to learn the complex and heterogeneous value cluster couplings in the high level. Different from existing representation methods, our work comprehensively captures the intrinsic data characteristic. Experiment results demonstrate that CDE++ is available for both supervised and unsupervised learning tasks, whereas it significantly outperforms existing state-of-the-art methods with good scalability and efficiency. Moreover, it is insensitive to its parameters.

Based on the superiority of CDE++, our future work is to consider mixed data (i.e., categorical and continuous data). Meanwhile, considering different applications requirements, we could customize CDE++ to get better performance.

## Figures and Tables

**Table 1:** A simple example to explain the value coupling relationships.

Name	Gender	Major	Occupation
John	Male	Engineering	Programmer
Tony	Male	Science	Analyst
Alisa	Female	Liberal arts	Lawyer
Ben	Male	Engineering	Programmer
Abby	Female	Liberal arts	Marketing Manager
James	Male	Engineering	Technician

**Table 2:** The descriptions of the notations in CDE++.

Symbols	Description
*X*, *x*	The dataset and a specific object.
*F*, *f*	The feature set in the dataset and a specific feature.
$f^{i}$	The feature that value $v_{i}$ belongs to.
*V*, *v*	The whole feature value set in the dataset and a specific feature value.
$V_{i}$	The feature value set for feature $f_{i}$ in the dataset.
$v_{x}^{f}$	The value in feature *f* of object *x*.
*n*	The number of objects in the dataset.
*d*	The number of features in the dataset.
*m*	The number of feature values in the dataset.
|C|	The number of groud-truth classes in the dataset.
p(v)	The probability of *v* that calculated by its occurrence frequency.
$p(v_{i},v_{j})$	The joint probability of $v_{i}$ and $v_{j}$.
$\rho (f_{a},f_{b})$	The relation between two features $f_{a}$ and $f_{b}$.
$I(f_{a},f_{b})$	The relative entropy of joint distribution and marginal distribution between two features $f_{a}$ and $f_{b}$.
H(f)	The marginal entropy of featrure *f*.
$\xi_{o}$	The occurrence-based value coupling function.
$\xi_{c}$	The co-occurrence-based value coupling function.
$M_{o}$	The occurrence-based relationship matrix.
$M_{c}$	The co-occurrence-based relationship matrix.
τ(eps,MinPts)	The parameter of DBSCAN.
*K*	The number of clusters parameter of HC.
*C*	The cluster indicator matrix.
vc	The dimension of cluster indicator matrix.
ε	The factor of drop redundancy value clusters.
λ	The hidden factor of Autoencoder.
*q*	The dimension of value after Autoencoder.
Ω	The general function to generate new objects embedding.

**Table 3:** The Dataset attributes and F-score results of Clustering by Inverse Document Frequency (IDF), DILCA, Categorical Data Embedding (CDE), CDE-AE, 1-HOT, 1-HOT-AE, and our method CDE++ on 15 Data Sets. The best performance for each data set is boldfaced. The Data Sets are sorted in descending order of F-score.

Dataset_Attributes					F-Score						
Datasets	n	d	m	|C|	IDF	DILCA	CDE	CDE-AE	1-HOT	1-HOT-AE	CDE++
Zoo	101	17	43	7	0.827	0.746	0.833	0.79	0.826	0.871	**0.879**
Iris	150	4	123	3	0.59	0.632	0.717	0.667	0.585	0.467	**0.8**
Hepatitis	155	19	360	2	0.535	0.679	0.672	0.687	0.677	0.684	**0.755**
Tic-tac-toe	958	9	27	2	0.536	0.542	0.557	0.559	0.578	0.578	**0.659**
Annealing	798	38	317	5	0.512	0.534	0.577	0.547	0.528	0.588	**0.654**
Bloger	100	5	15	2	0.484	0.492	0.546	0.539	0.53	0.51	**0.61**
Balance-scale	625	4	20	3	0.463	0.497	0.514	0.499	0.462	0.419	**0.6**
Lymphography	148	18	59	4	0.556	0.513	0.528	0.489	0.494	0.493	**0.568**
Hayes-roth	132	4	15	3	0.48	0.478	0.495	0.484	0.348	0.341	**0.545**
Teaching A.E.	151	5	101	3	0.395	0.41	0.432	0.44	0.428	0.444	**0.503**
Student A.P.	131	21	75	3	0.429	0.423	0.449	0.433	0.445	0.466	**0.475**
Lenses	24	4	9	3	0.442	0.471	0.458	0.467	0.546	**0.583**	0.458
Nursery	12,960	8	27	5	0.306	0.294	0.32	0.356	0.283	**0.382**	0.325
Primary-tumor	339	17	37	21	0.218	0.224	0.285	0.29	0.291	0.289	**0.299**
Chess	28,056	6	40	18	0.154	0.16	0.165	0.16	0.157	0.151	**0.174**
**Average**					0.462	0.473	0.503	0.494	0.479	0.484	**0.554**

**Table 4:** Basic Parameters of Autoencoder.

Architecture ${}^{†}$	A-64-code dimension-64-$A^{\prime }$
MaxEpochs	1000
LossFunction	MSE with L2 and Sparsity Regularizers
Training Algorithm	Scaled Conjugate Gradient Descent

${}^{†}$ The code dimension in Architecture is determined by the hidden factor λ and the original data dimension.

**Table 5:** The Accuracy results of Classifying by IDF, DILCA, CDE, CDE-AE, 1-HOT, 1-HOT-AE, and our method CDE++ on 15 Data Sets. The best performance for each data set is boldfaced. The Data Sets are sorted in descending order of Accuracy.

	Accuracy						
Datasets	IDF	DILCA	CDE	CDE-AE	1-HOT	1-HOT-AE	CDE++
Zoo	0.937	0.946	0.97	0.944	1	1	1
Lenses	0.826	0.793	0.833	0.714	0.8	0.811	**1**
Annealing	0.973	0.979	0.985	0.978	**0.991**	0.989	0.988
Tic-tac-toe	0.894	0.872	0.913	0.735	0.981	0.98	**0.984**
Balance-scale	0.741	0.713	0.727	0.649	**0.968**	0.957	**0.968**
Nursery	0.804	0.729	0.817	0.549	0.937	0.939	0.943
Iris	0.883	0.897	0.893	0.887	0.92	**0.927**	0.911
Bloger	0.742	0.733	0.739	0.808	0.758	0.852	**0.903**
Hepatitis	0.829	0.817	0.834	0.811	0.877	0.804	**0.894**
Hayes-roth	0.754	0.771	0.807	0.763	0.829	0.834	**0.861**
Lymphography	0.792	0.803	0.819	0.803	0.826	0.834	**0.849**
Primary-tumor	0.538	0.551	0.577	0.528	0.595	0.61	**0.623**
Student A.P.	0.518	0.499	0.529	0.519	0.551	0.544	**0.615**
Teaching A.E.	0.547	0.543	0.576	0.509	0.561	0.585	**0.613**
Chess	0.184	0.216	0.242	0.215	0.27	0.257	**0.413**
**Average**	0.731	0.724	0.751	0.694	0.791	0.795	**0.838**

**Table 6:** Ablation Study Settings.

	Learning Value Clusters		Learn Value Clusters Couplings
	DBSCAN	HC	Autoencoder
i	🗸	×	×
ii	×	🗸	×
iii	🗸	🗸	×
iv	🗸	🗸	🗸

**Table 7:** F-score Results of Ablation Study based on Clustering task. The best performance for each data set is boldfaced.

	F-Score			
Datasets	DBSCAN	HC	DBSCAN+HC	CDE++
Zoo	0.829	0.85	0.854	**0.879**
Iris	0.453	0.491	0.646	**0.8**
Hepatitis	0.503	0.617	0.666	**0.755**
Tic-tac-toe	0.621	0.578	0.573	**0.659**
Annealing	0.606	0.514	0.544	**0.654**
Bloger	0.53	0.588	0.572	**0.61**
Balance-scale	0.464	0.5	0.549	**0.6**
Lymphography	0.434	0.488	0.484	**0.568**
Hayes-roth	0.445	0.445	0.441	**0.545**
Teaching.A.E	0.437	0.419	0.491	**0.503**
Student.A.P	0.435	0.425	0.446	**0.475**
Lenses	**0.625**	0.583	0.583	0.458
Nursery	**0.333**	0.295	0.273	0.325
Primary-tumor	0.248	0.291	0.288	**0.299**
Chess	0.163	0.167	0.151	**0.174**
**Average**	0.475	0.483	0.504	**0.554**

**Table 8:** Accuracy Results of Ablation Study based on Classifying task. The best performance for each data set is boldfaced.

	ACCURACY			
Datasets	DBSCAN	HC	DBSCAN+HC	CDE++
Zoo	0.964	0.97	0.976	**1**
Lenses	**1**	0.789	0.811	**1**
Annealing	0.975	0.93	0.971	**0.988**
Tic-tac-toe	0.695	0.965	0.977	**0.984**
Balance-scale	**0.971**	0.961	0.958	0.968
Nursery	**1**	0.944	0.943	0.943
Iris	0.833	0.738	0.898	**0.911**
Bloger	0.839	0.781	0.819	**0.903**
Hepatitis	0.828	0.838	0.845	**0.894**
Hayes-roth	0.812	0.837	0.834	**0.861**
Lymphography	0.828	0.813	0.843	**0.849**
Primary-tumor	**0.625**	0.579	0.564	0.623
Student.A.P	0.578	0.527	0.527	**0.615**
Teaching.A.E	0.565	0.557	0.578	**0.613**
Chess	**0.665**	0.461	0.236	0.413
**Average**	0.812	0.779	0.785	**0.838**
